# Transdermal Polymeric Microneedle Sensing Platform for Fentanyl Detection in Biofluid

**DOI:** 10.3390/bios12040198

**Published:** 2022-03-27

**Authors:** Pratik Joshi, Parand R. Riley, Rupesh Mishra, Sina Azizi Machekposhti, Roger Narayan

**Affiliations:** 1 Department of Materials Science and Engineering, UNC/NCSU Joint Department of Biomedical Engineering, NC State University, Raleigh, NC 27695, USA; pjoshi5@ncsu.edu (P.J.); prostam@ncsu.edu (P.R.R.); sazizim@ncsu.edu (S.A.M.); 2 Identify Sensors Biologics, 1203 W. State St., West Lafayette, IN 47907, USA; rupeshmishra02@gmail.com

**Keywords:** microneedle, fentanyl, serum, ionic liquid, opioid, electrochemical detection

## Abstract

Opioid drugs are extremely potent synthetic analytes, and their abuse is common around the world. Hence, a rapid and point-of-need device is necessary to assess the presence of this compound in body fluid so that a timely countermeasure can be provided to the exposed individuals. Herein, we present an attractive microneedle sensing platform for the detection of the opioid drug fentanyl in real serum samples using an electrochemical detection method. The device contained an array of pyramidal microneedle structures that were integrated with platinum (Pt) and silver (Ag) wires, each with a microcavity opening. The working sensor was modified by graphene ink and subsequently with 4 (3-Butyl-1-imidazolio)-1-butanesulfonate) ionic liquid. The microneedle sensor showed direct oxidation of fentanyl in liquid samples with a detection limit of 27.8 μM by employing a highly sensitive square-wave voltammetry technique. The resulting microneedle-based sensing platform displayed an interference-free fentanyl detection in diluted serum without conceding its sensitivity, stability, and response time. The obtained results revealed that the microneedle sensor holds considerable promise for point-of-need fentanyl detection and opens additional opportunities for detecting substances of abuse in emergencies.

## 1. Introduction

Fentanyl is a prevailing synthetic opioid (schedule II) drug that is analogous to morphine but almost 100 times more potent [1]. This prescription drug is also made and used in an illicit manner. Similar to morphine, it is a medicine that is typically used to treat patients with severe pain, particularly after surgery. The United States has been experiencing an epidemic related to opioid use disorder, resulting in ∼100 American deaths per day due to opioid overdose; this epidemic is associated with a monetary impact of ∼$100 B/year [2]. Newborns are some of the many victims of this epidemic due to the usage of opioids during pregnancy, resulting in addicted infants who can suffer from Neonatal Abstinence Syndrome (NAS) [3]. There are also growing concerns concerning the potential use of opioids by terrorists and others in civilian settings. For example, the application of fentanyl drugs during the 2002 hostage situation in Moscow resulted in over 100 deaths [4]. These agents can be absorbed by the skin and cross-cutaneous membranes. Opioids represent a grave threat to public health and safety to first responders since even small amounts of opioids present can become aerosolized and toxic [5]. In 2015, the Drug Enforcement Agency declared a nationwide warning calling fentanyl a “threat to health and public safety.” Moreover, recently the Centers for Disease Control and Prevention reported that fentanyl and associated analogs related to over half of the opioid overdoses in ten states during the second half of 2016 [6]. Thus, the detection of fentanyl in trace quantities is essential to dealing with the current widespread epidemic. Hence, there is an urgent need for the rapid identification of exposure to such chemical threats for lifesaving therapeutic intervention. Wearable devices could transform the field of drug addiction by delivering point-of-need rapid detection [6]. Even though these initial devices offer valuable knowledge for point-of-need detection of suspicious powders, the continuous post-exposure on-body tracking of these threats has not been explored to this point.

Small-scale devices with micro and nanoscale features are the inevitable future of sensor technology [7,8]. These devices have been used in various configurations and types such as nanowire field-effect transistors [9], microcantilevers [10], optical micro-ring resonators [11], and micro and nanoneedles [6,12]. Among them, micro and nanoneedles conjugated with different analytical methods, including voltammetry, amperometry, and intravital microscopy, are widely utilized for biosensing applications [13]. Most of the detection methods require the capillary collection of the interstitial fluid, micro pumping of the interstitial fluid, or a proper surface binding between the device and the target biomolecule [14,15,16,17]. In situ selective detection of glucose, lactate, glutamate, ascorbic acid, and hydrogen peroxide has been possible with integrated microneedle-electrode devices [18]. Bollella et al. reported pain-free microneedles for the continuous detection of lactate in the dermal interstitial fluid [3]; they functionalized the surface of gold microneedles with carbon nanotubes and methylene blue and demonstrated real-time lactate sensing. In another study, Zhao et al. performed the continuous detection of glucose by a microneedle conjugated with an electrochemical method [19]. They used a silk/polyols/glucose-oxidase microneedle for the long-term, minimally invasive sensing of glucose. Goud et al. developed a wearable electrochemical microneedle for the continuous monitoring of levodopa (L-Dopa) [20]; L-Dopa is an important medication for Parkinson’s disease management. The multimodal microneedle system evaluated by Goud et al. consisted of a microneedle array with orthogonal electrochemical and biocatalytic components.

The fabrication of microneedles plays a vital role in the performance of the small-scale biosensing device. Hollow needles provide an ideal platform for transdermal biosensing and drug delivery [21]. Microneedles with lengths between 480–1450 µm cause less pain compared to 26 gauge hypodermic needles, as less contact arises between microneedles and Pacinian corpuscles [22,23]. Microneedles can be fabricated with a plethora of materials such as glass, Si, stainless steel, and polymers [24,25,26,27]. The earliest microneedles were made from Si due to their compatibility with microelectronics. However, the brittle nature of Si was a limiting factor of Si use in microneedle fabrication. However, the microneedles synthesized by conventional methods have not been widely employed in clinical use. In this study, microneedles were fabricated with E-Shell 200, which is a Class-IIa biocompatible, water-resistant material. Studies have confirmed the E-Shell 200 microneedles fabricated using two-photon polymerization and polydimethylsiloxane micromolding processes were successfully able to penetrate the human stratum corneum and epidermis [28]. E-Shell 200 made by Envisiontec (Gladbeck, Germany) is a photoreactive polymer consisting of biocompatible elements such as carbon, titanium, and oxygen. The detailed composition of this material is provided in Table 1 [29]. 

E-Shell 200 is characterized by low water absorption (0.12%) and a high glass transition temperature of 109 C; flexural- and tensile-strength values of 57.8 and 108 GPa, respectively, for this material have been described [19,20]. In addition, this material demonstrates high hardness and Young’s modulus values of ~94 MPa and ~3.05 GPa, respectively [30]. Per Park et al., this high Young’s modulus value suggests that the fracture forces of E-Shell 200 are greater than typical skin-insertion forces [25]. E-Shell 200 microneedles have been successfully employed to detect changes in pH, lactate, and glucose [31].

In this work, we present the applicability of a 3D-printed microneedle array to detect fentanyl in serum and demonstrate a novel sensing platform for screening opioids in real biofluid. Microneedles have recently earned enormous interest owing to their capability to continuously examine a wide scale of clinically significant analytes. Several important analytes and biomarkers have recently been detected using different microneedle sensing methods. 

A hollow microneedle platform is explored for fentanyl detection, which was prepared by 3D printing E-Shell 200 material. Subsequently, platinum, silver, and graphene ink were inserted into the hollow microneedle junctions. Graphite ink is used extensively for the construction of biosensors due to the low background current and the capability for surface regeneration [32]. Graphene ink modified using ionic liquid 4 (3-butyl-1-imidazolio)-1-butanesulfonate) was used as a working sensor, the platinum electrode was used as a counter electrode, and silver wire was inserted and used as a reference-electrode system. After the preparation of this microneedle setup, fentanyl concentrations between 0–160 µM were tested in a buffer solution with good linearity and detection limit. Furthermore, the microneedle sensor was extended to detect fentanyl in diluted serum samples. Good analytical performance was obtained with satisfactory repeatability and limit of quantification. The microneedle sensor platform modified using 4 (3-Butyl-1-imidazolio)-1-butanesulfonate) liquid could serve as an effective sensing platform for rapid fentanyl-screening applications in a real biofluid. 

## 2. Materials and Methods

### 2.1. Chemicals 

Fentanyl was mixed in 1.0 mg/mL of methanol; graphene ink was used to modify the working-electrode surface. Other chemicals (e.g., ascorbic acid, uric acid, glucose, phosphate buffer, and ethanol) were all purchased from Sigma-Aldrich (St Louis, MO, USA). The bovine plasma in the Na-Citrate (filtered) sample was procured from Lampire Biological Laboratories (Pipersville, PA, USA). All of the solvents and other chemicals used in this study were of analytical grade and were used without any further processing.

### 2.2. Microneedle Fabrication

Computer-aided design drawings were used to fabricate the microneedle structure [1,2]. This low-cost approach does not require the use of cleanroom facilities [3]. Hollow microneedle arrays were fabricated with three-dimensional drawings by Solidworks (Dassault Systèmes, Paris, France). The microneedle bases were triangular with 1.2 mm sides, a height of 1.6 mm, and a vertical cylinder channel diameter of 400 μm. Three needles were arranged in a line with 2 mm interspacing. The substrate was a circle with 8 mm diameter and a thickness of 1 mm. The microneedle-array-manufacturing process was performed by a Perfactory III SXGA instrument EnvisionTEC GmbH, Gladbeck, Germany. E-shell 200 resin was used as a photopolymer that was polymerized by a 150 W halogen bulb as a light source. Selective polymerization of the material was performed in a layer-by-layer manner using a digital micromirror device (Texas Instruments, Dallas, TX, USA). It has a build envelope of 90 mm * 67.5 mm. After finishing the E-shell polymerization by the EnvisionTEC printer, the array was soaked in isopropanol and sonicated for removal of the unpolymerized materials. Post-processing was performed using an Otoflash instrument (EnvisionTEC GmbH, Gladbeck, Germany). It has two photoflash lamps that provide a wavelength light range of 300–700 nm. The microneedle-array structure was assessed using an S3200N variable-pressure scanning electron microscope (Hitachi, Tokyo, Japan). The microneedle height was assessed using a VKx1100 laser scanning microscope (Keyence, Tokyo, Japan).

### 2.3. Electrochemical Characterization 

The electrochemical operation for fentanyl detection was conducted using a polymeric microneedle platform that had three electrodes: platinum as a counter electrode, silver as a reference electrode, and graphene-ink-modified platinum wire as a working electrode. The SWV studies were performed on a microneedle platform using 0.1 M PBS solution at 7.4 pH at room temperature by swiping the electrode potential (vs. Ag) between 0 and +0.9 V. The fentanyl stock solutions were prepared in methanol and were diluted in PBS solution using the successive dilution method and were used for electrochemical analysis. Before testing the microneedles, the sensor platform was cleaned with ethanol and distilled water and subsequently air dried. 

**Safety note:** Fentanyl is a very powerful synthetic and potentially lethal opioid even at extremely low levels as 0.25 mg according to the Drug Enforcement Administration (DEA) [33]. Additionally, fentanyl may be absorbed via the skin or accidentally inhaled with associated health implications. Thus, extreme caution is to be practiced while handling these drugs; all chemical stock preparation was conducted in a fume hood with appropriate Personal Protective Equipment (PPE) in accordance with the university protocol.

## 3. Results

### 3.1. Fentanyl Detection on Microneedle Surface

**Figure 1:**
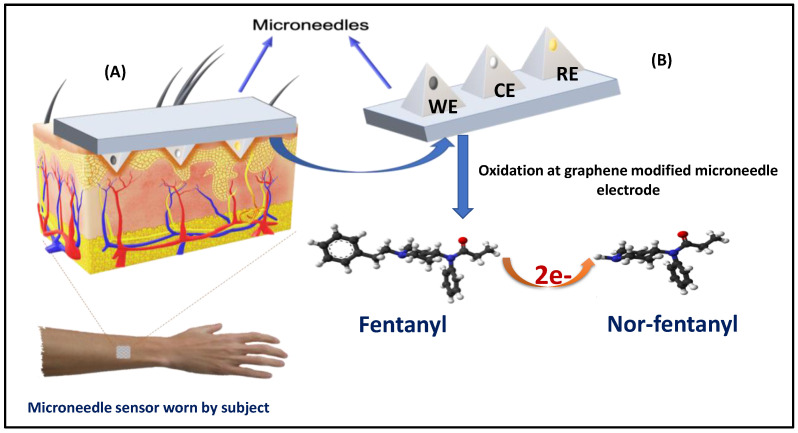
Schematic illustration of fentanyl detection on graphene-modified microneedle working sensor and sensor detection mechanism for future on-body applications. (**A**) Microneedle array placed on the skin for fentanyl sensing and (**B**) fentanyl oxidation at graphene-modified microneedle sensor using SWV.

The direct voltammetric detection of fentanyl was conducted on a working sensor modified by graphene ink and subsequently with 4 (3-butyl-1-imidazolio)-1-butanesulfonate) ionic liquid. The ionic liquid plays an important role in increasing the conductivity and also acts as a strong electron-shuttling agent. The graphene-modified microneedle working electrode involves an oxidative dealkylation of the piperidine tertiary amine and nor-fentanyl structure, which exhibits a unique single peak at +0.55 V upon rhythmic scans reflecting the two-electron oxidation of the fentanyl tertiary amine groups. The obtained baseline-subtracted calibration plot confirms the linearity of this anodic current response with the fentanyl dosage over the tested range. Figure 1 illustrates the principle of fentanyl detection on a graphene-modified microneedle sensor.

**Figure 2:**
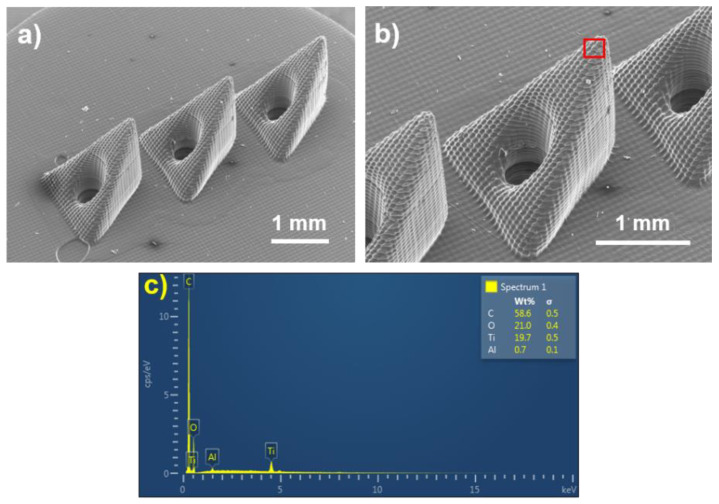
(**a**) Low-magnification SEM image of the microneedle array, showing all three tips, (**b**) high-magnification image of a single microneedle within the array, and (**c**) EDS data from the microneedle tip (red rectangular region) in (**b**).

Although previous fentanyl sensors have been described as reported using screen-printed electrodes [15,16], glove platforms, microneedles, and standard electrodes [17], this is the first report that describes a platinum-based sensor modified by graphene ink to detect fentanyl levels in serum. Figure 2a,b show scanning electron micrographs of the microneedle array; Figure 2c shows the energy-dispersive-X-ray-spectroscopy (EDS) results from the microneedle. The EDS data indicate that the microneedle material is mostly composed of carbon (58.6 wt.%) and some oxygen (21 wt.%), titanium (19 wt.%), and a small amount of aluminum. The microneedle height was assessed using a VKx1100 laser scanning microscope (Keyence, Tokyo, Japan) to be 1.63 mm.

**Figure 3:**
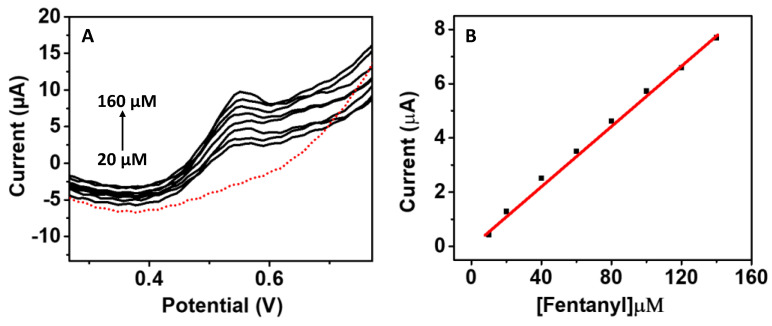
Calibration curve of fentanyl in buffer solution using microneedle sensor platform. Figure (**A**) illustrates SWV response of increasing fentanyl (20–160 μM, each 20 μM addition) concentrations in buffer solution; the red dotted curve presents buffer baseline current response and other black curves illustrate the fentanyl oxidation curve on polymeric microneedle surface. Figure (**B**) shows the obtained current response (baseline subtracted) from the fentanyl calibration (20–200 μM). The SWV parameters were an equilibrium time of 2 s, a potential range of 0–1.0 V, an E step of 0.01 V, and a frequency of 10 Hz; the experiments were performed at room temperature.

The electron-transfer characteristics and the earlier-reported carbon-chain conductivity were observed with microneedle electrodes using a 1 mM ferro/ferricyanide solution. The obtained CV peak showed a good response with the redox probe; therefore, we conducted the tests with fentanyl solution. Figure 3A illustrates a fentanyl calibration obtained in buffer solution. The calibration curve is important to establishing the detection of any analytes and provide a reference point to compare the obtained data in real samples. The microneedle sensor was tested with fentanyl over a wide concentration range between 20–160 μM (Figure 3B). Initially, fentanyl stock solution (1000 ppm) was diluted to 1000 µM in water; the solution was further diluted to a sub stock solution of 200 µM. From this sub stock solution, 10 µL was dispensed on the microneedle surface and mixed with buffer to make up the 100 µL total volume. A sequencial additon of 20 µL of fentanyl was performed until the sensor reached a saturation point. A well-identified peak of fentanyl oxidation was observed at +0.55 V, signifying the two-electron oxidation from the analyte’s (fentanyl) tertiary amine groups. The resultant calibration graph shows a linear response of this anodic current response with the drug concentration over the tested concentration range. In previous reports of fentanyl detection, the oxidation peak of fentanyl was obtained around +0.7 V using graphene ink and ionic liquid. The incorporation of the graphene ink helped in providing a conductive surface with an appropriate surface area on the sensing electrode for fentanyl detection; the ionic liquid 4 (3-butyl-1-imidazolio)-1-butanesulfonate) significantly enhanced the signal detection. The initial testing of fentanyl in buffer solution confirmed that the graphene-modified Pt can be utilized for drug detection in the liquid phase and warranted testing in real biofluid samples. The obtained linear range showed a good sensitivity of 0.054 µA/µM with a relative standard deviation (RSD) of 3.1%. The experiments were perfomed at least three times to ensure good reproducibility among the tested sensors. Origin software 5.8 (OriginLab Corporation, Northampton, MA, USA) was used for data analysis, sensitivity, and % RSD determination. The calculated LOD for fentanyl in buffer solution was 27.8 µM, with a standard deviation below 1.

### 3.2. Fentanyl Detection in Serum Sample

**Figure 4:**
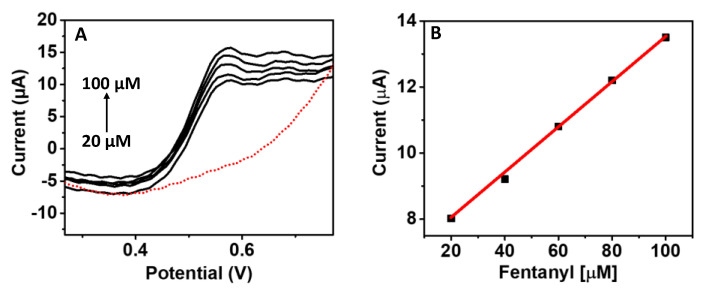
Application of microneedle sensor for fentanyl sensing in a real biofluid. (**A**) SWV response of increasing fentanyl (20–100 μM) concentrations with each 20 μM increase in the diluted serum samples (**B**) The corresponding current response from the fentanyl-detection curve (**A**). The SWV parameters were an equilibrium time of 5 s, potential range 0–0.9 V, E step 0.01 V, frequency 15 Hz; the experiments were performed at room temperature.

To further extend the applicability of the developed microneedle for fentanyl detection, the sensor was examined in real serum samples by the dosing method wherein varying fentanyl concentrations between 20–100 μM (in 20 μM increments) were spiked in the serum sample and tested on the microneedle sensor. Several sensors have been employed to identify the concentration of fentanyl in liquid samples such as saliva and blood; however, this is the first report of fentanyl diagnostics using a microneedle sensor in serum. The serum samples were commercially procured and directly used for electrochemical testing. The raw serum samples were spiked with fentanyl and tested; however, the level of fentanyl was not detected on the microneedle sensors; therefore, the serum samples were diluted to five times and again tested after spiking with fentanyl. In the diluted samples of serum, the oxidation peak of fentanyl could be detected. As presented in Figure 4, the microneedle sensor exhibited a similar sensing pattern to that observed in the 0.1 M PBS solution. Good linearity was observed in the serum sample for fentanyl testing with an R^2^ = 0.99 (*n* = 3), a sensitivity of 0.069 µA/µM, and an LOD (limit of detection) of 29.2 µM.

Such unique analytical performance indicates an encouraging perspective on fentanyl detection using microneedles, and could potentially serve as a point-of-need detection for first responders and soldiers in opioid detection. The fentanyl oxidation peak is slightly shifted with the increase in analyte concentration due to the accumulation of the oxidation product on the surface, and possibly due to surface fouling. It is important to mention that the reaction takes place in a small surface area. This phenomenon will certainly affect the analysis when considering real samples with equipment that is optimized for use outside the laboratory. Future efforts will involve utilizing a printed circuit board and integrating the microneedle biosensor for a digitalized output in which the the increase in current value will be displayed.

### 3.3. Sensor Selectivity

**Figure 5:**
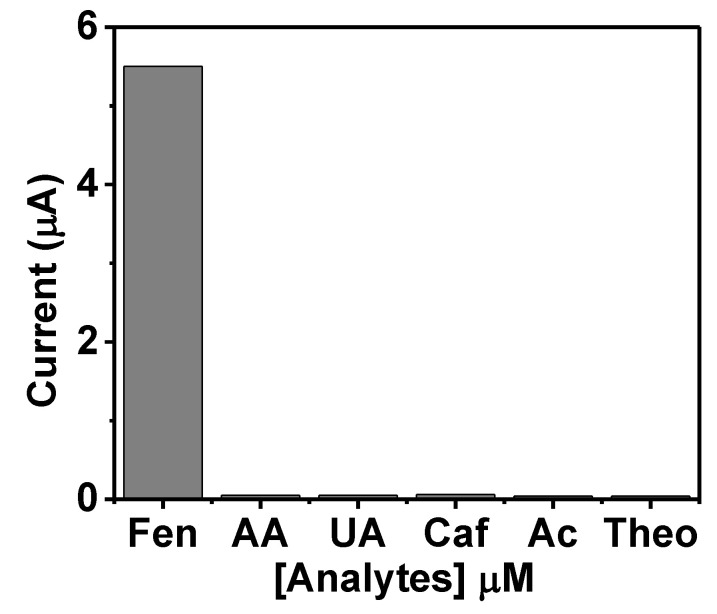
The selectivity performance of polymeric microneedle-based fentanyl sensor toward prospective interfering analytes. The bar graph illustrates the current values obtained by SWV response of fentanyl (50 mM), ascorbic acid, uric acid, caffeine, acetaminophen, and theophylline (all these tested interfering analytes were at the concentrations of 1000 mM).

The selectivity of the sensor is important for distinguishing the analyte of interest in the presence of other similar co-existing molecules. Interferents such as uric acid, caffeine, ascorbic acid, acetaminophen, and theophylline were tested along with fentanyl as their presence in biofluid is possible. Ideally, a sensitive and reliable sensor should be able to differentiate the target analyte from interfering agents. Figure 5 illustrates the developed selectivity performance for fentanyl by recording the SWV of the transdermal polymeric microneedle surface with 1000 μM ascorbic acid, uric acid, caffeine, acetaminophen, theophylline, and fentanyl (50 μM). A noticeable oxidation peak at +0.55 V corresponding to the fentanyl was noted in the cyclic voltammograms, which was found to be reduced in the presence of the interferents, but not significantly. No visual current drift due to possible interferences was observed, validating the selectivity performance towards fentanyl. The results reveal that the tested interfering elements do not affect fentanyl detection. These results show that the microneedle sensor can be very selective in fentanyl screening. The analytical performance of the developed sensor supports the use of the microneedle sensor for possible use in fentanyl point-of-need detection. The sensor-selectivity test was repeated three times.

## 4. Conclusions

This work presented the development of a microneedle platform for the minimally invasive detection of fentanyl in serum. The microneedle sensor array was able to distinguish between different fentanyl concentrations, which enables the potential application of this technology to medical diagnosis. Additionally, the new microneedle-sensor-array platform showed an attractive analytical response in the tested serum samples with high sensitivity, selectivity, and stability. The dynamic range of fentanyl detection was observed between 20–160 μM, with an LOD of 27.8 µM. This platform has the potential for use by first responders, clinicians, and on-site soldiers for rapid detection of fentanyl. 

## Figures and Tables

**Table 1:** Materials used to synthesize E-Shell 200 [30].

Material	wt.%
Phenylbis(2,4,6 trimethylbenzoyl)-phosphine oxide photoinitiator	0.5–1.5
Propylated (2) neopentyl glycoldiacrylate	15–30
Urethane dimethacrylate	60–80

## Data Availability

The data presented in this study are available on request from the corresponding author.
